# Window dressing: possibilities and limitations of incremental changes in solitary confinement

**DOI:** 10.1186/s40352-021-00145-7

**Published:** 2021-07-31

**Authors:** Dallas Augustine, Melissa Barragan, Kelsie Chesnut, Natalie A. Pifer, Keramet Reiter, Justin D. Strong

**Affiliations:** 1 Department of Medicine, University of California, San Francisco, USA; 2 Department of Sociology, California State Polytechnic University at Pomona, Pomona, USA; 3 Vera Institute of Justice, Brooklyn, USA; 4 Department of Criminology and Criminal Justice, University of Rhode Island, Kingston, USA; 5 Department of Criminology, Law & Society, University of California, Irvine, USA

**Keywords:** Reform, Segregation, Policy, Corrections, Correctional health

## Abstract

**Background:**

In light of mounting evidence of the physical and psychological harms associated with solitary confinement, many correctional systems, state legislators, courts, and even international human rights bodies are increasingly recommending and implementing reforms to mitigate the harms of solitary confinement, if not abolish the practice entirely. In this piece, we examine three specific infrastructural changes to solitary confinement conditions and practices implemented in Washington state prisons with such harm minimization goals in mind: (1) building so-called “nature imagery rooms” to play videos of outdoor spaces, (2) eliminating punishments for self-harm, and (3) conducting daily cell-front wellness checks.

**Results:**

Drawing on 183 in-depth qualitative interviews with both staff working in and people imprisoned in solitary confinement units conducted in Washington state restrictive housing units in 2017, we find that these three reforms not only resulted in limited successes but also generated new conflicts. Institutional logics such as deprivation, risk-management, and responsibilization ultimately impeded even the most modest attempts to mitigate the inherently harsh practice of solitary confinement. The limits of these reforms are due in part to individual choices made by people imprisoned in solitary confinement and staff working in these units, as well as the larger cultural norms that shape life in restrictive housing units.

**Conclusions:**

Incrementalist reforms aimed at softening the environment of solitary confinement may actually serve to increase the strain and stress experienced by people confined to and working within them. Even the most well-intentioned reforms, like those attempted by the Washington DOC, should be scrutinized in order to determine if they are producing the desired outcomes, or instead, reproducing a different, but nonetheless damaging set of harms to people imprisoned in solitary confinement. Further, even well-intentioned reforms are often stymied by the underlying institutional logics of restrictive housing spaces.

Every year, tens of thousands of people imprisoned in U.S. prisons, jails, and immigrant detention centers experience solitary confinement – locked in cells the size of wheelchair-accessible bathroom stalls, where always-on fluorescent lights replace windows, and where opportunities for human contact are limited for days, weeks, months, and sometimes years on end (Association of State Correctional Administrators and the Arthur Liman Public Interest Program, Yale Law School (ASCA-Liman), 2018; Beck, 2015; Franco et al., 2020). The dangerous psychological effects of general social isolation are well-documented, and an increasingly robust literature has established the psychological as well as physical harms of solitary confinement: anxiety, depression, psychosis, skin irritations, brain atrophy, cardiovascular disease, and even increased mortality risk (Haney, 2020; Lobel & Akil, 2018; Reiter et al., 2020; Stahn et al., 2019; Strong et al., 2020; Williams et al., 2019; Wildeman & Andersen, 2020; Zigmond & Smeyne, 2020). In light of such mounting evidence, many correctional systems, state legislators, courts, and even international human rights bodies are increasingly recommending and implementing reforms to mitigate the harms of solitary confinement, if not abolish the practice entirely (Haney, 2018; Reiter, 2018; Schlanger, 2020). Indeed, the American Institute of Architects (AIA) has recently revised their Code of Ethics and Professional Conduct to prohibit members from designing spaces for solitary confinement as it conflicts with the AIA’s values of safety, welfare, and human rights (American Institute of Architects, 2020).

In this piece, we examine three specific infrastructural changes to solitary confinement conditions and practices implemented in Washington state prisons: (1) building so-called “nature imagery rooms” to play videos of outdoor spaces, (2) eliminating punishments for self-harm, and (3) conducting daily cell-front wellness checks. These changes were implemented over the past few years in the state’s five Intensive Management Units (IMUs), which house Washington’s highest security population. Each of the three changes was implemented as part of a statewide correctional effort to both limit solitary confinement use and mitigate the harsh conditions and health harms of solitary confinement when imposed (Washington Department of Corrections, 2020a). This paper utilizes data from fieldwork conducted in Washington IMUs in 2017. Drawing on 183 in-depth qualitative interviews with both staff working in and people imprisoned in solitary confinement^1^ units, we analyze how people living and working in these units experienced these reforms. Through this analysis, we identify both the possibilities and limits of reform in the highly restrictive environment of solitary confinement units (specifically IMUs in Washington state), unpacking the relationship between the built environment of a prison, institutional logics, correctional policy, and lived experience.

## Background

Our examination of three infrastructural changes to solitary confinement in Washington state implicates and integrates two theoretical frameworks for understanding whether and how reform agendas succeed in improving conditions of confinement: one centered in carceral geography and another in institutional logics. Here we provide a brief overview of both frameworks and how they deepen our analysis of reform in the context of solitary confinement specifically. Ultimately, we argue that attending to the lived experience of infrastructural reforms in solitary confinement, as carceral geographers call for (Jewkes, 2018; Moran, 2013), reveals how space shapes institutional logics, but also how institutional logics, in turn, constrain infrastructural reforms.

Carceral geography “foreground [s] the experience of carceral space, both in terms of the individual’s movement into and out of that space and his or her experience within it” (Moran, 2013: 175). This focus on space builds on Foucault’s observation that contemporary forms of punishment entail a particular kind of space-making (Foucault, 1995). Imprisoned people are distributed within enclosed spaces arranged by techniques of supervision, individualization, and discipline. Such disciplinary regimes, in part, produce power through the overlay of spatial design and institutional logics. Ross succinctly calls this the “architecture of authority” (Ross, 2007), and Jewkes identifies the exact message of control communicated by this authoritative architecture: “cage-like interiors, bolted-to-the-floor furniture and vandal-resistant surfaces,” explicitly “communicates to prisoners” that they are “animals” and “potential vandals” (Jewkes, 2018: 321).

If this penal aesthetic is true of prison architecture generally, it is all-the-more-true of solitary confinement settings, even in reform-oriented places like Washington’s IMUs, where people are still housed alone in stark cells for as many as 22 h a day, and provided little human contact to distract from the implicit negative messaging about being an animal or a vandal. In such an environment, even a subtle mitigation of the harshness of the conditions – like an extra hour per day out of the cell, or time in the nature room, or an extra cell-front check-in – might represent both a significant improvement in imprisoned people’s day-to-day experiences and an explicit acknowledgement of their humanity (Reiter, 2014; Reiter & Blair, 2018). Indeed, Jewkes argues that a well-designed correctional institution, like a well-designed hospital, might allow an imprisoned person, like a patient, “to flourish” (Jewkes, 2018: 329). Softening the harsh “elements that make custodial institutions barren environments that break people” both “communicates positive attributes” to individual imprisoned people and challenges “the cultural stereotype of what a prison is” (Jewkes, 2018: 329, 334). Here, Jewkes recommends replacing barred and barren institutions with “large bar-less windows,” in “humane, sensuous, architecturally innovative facilities that go well beyond simply avoiding an institutional feel” (Jewkes, 2018: 329). Jewkes’s focus on softening presents a provocative but fundamental question for solitary confinement reform: Can a solitary confinement unit be softened to facilitate flourishing rather than suffering?

Our research suggests that, although incrementalist reforms, like the nature room or the cell-front check, force obvious spatial re-configurations, institutional logics of deprivation, risk-management, and responsibilization undermine the potential softening effects of these reformist reconfigurations. Indeed, while institutional logics are much less visible than the architectural attributes of a solitary confinement unit, they nonetheless can function as an obstacle to reform. Institutional logics, defined as the frame of reference by which organizational actors make meaning, motivate action, and shape identity (Thorton et al., 2012), have been identified as a key factor mediating reform efforts in prison settings in particular (Borchert, 2016; McPherson & Sauder, 2013; Rudes et al., 2020; Rubin, 2019).

For instance, Rudes et al. examine how implementation of the Prison Rape Elimination Act (PREA) produced a competition between new logics of imprisoned people’s inherent dignity and “existing logics/cultures” of control, mistrust, and hypermasculinity (Rudes et al., 2020: 5). Rudes et al. found that male correctional staff often perceived efforts to eliminate sexual violence as burdensome and ineffective, and in resisting implementation of PREA, staff reinforced (rather than softened) institutional logics of hypermasculinity and adversariality. In another analysis of the role of pre-existing logics and cultures in thwarting prison reform, Rubin posits the idea of legal templates: “idealized, replicable models that specify, in varying degrees of detail, punishment’s structure – what punishment should look like, of what activities it would consist, who is involved, where it takes place, or how long it lasts” (Rubin, 2019: 526). Like institutional logics, legal templates can “become[] so well ensconced in the penal landscape that, even when the template seems a poor fit for the challenges at hand, jurisdictions create new versions of the template rather than seeking altogether different templates” (Rubin, 2019: 539). Solitary confinement is, arguably, a core aspect of the legal template of punishment, and one that persistently appears and re-appears through the very process of tinkering with the spatial designs and institutional logics of the practice itself.

As both Rubin and Rudes document, prisons resist reform, especially reform of the incrementalist variety. An incrementalist or reformist reform is a “partial or ameliorative” measure, as compared to maximalist or non-reformist reforms which tend to be “more thoroughgoing,” seeking systematic re-structuring, by, say, defunding the police, or abolishing solitary confinement entirely (Schlanger, 2020: 275). Efforts at incorporating “green” prison design elements are also incremental in how they aim to “transform” but nonetheless keep in place the prison itself. The three reforms we examine are clearly incremental, seeking to mitigate, not abolish, the harsh conditions of solitary confinement. Through our analysis of interviewee responses to infrastructural IMU reforms in Washington state (see Table 1 in Appendix A for a list), we ask whether and how incrementalist reforms might be sustained and lead to more systemic change (Mathiesen, 2014; Schlanger, 2020).

By analyzing the implementation of Washington IMU reforms, we are able to assess whether these interventions have had or might have more transformative effects on either the prison environment or the institutional logics and legal templates governing that environment. Indeed, Jewkes and Moran acknowledge that while the incorporation of ‘green’ technology into new prison builds and retrofits … may deliver some genuine gains,” reformers also risk justifying continued investments in carceral infrastructure and/or distracting from the harms that such infrastructure can generate and impose (Jewkes & Moran, 2015: 15). Likewise, in the case of reforms within long-term solitary confinement units, the “gains” of softening the everyday experience of solitary confinement have to be weighed against the possibility that these efforts may also refine and reinforce the practice of solitary confinement, as well as make the potential harms of the conditions harder to see and challenge (Pifer, 2016; Reiter, 2012).

Interestingly, “there has been relatively little research on how institutional design is actually experienced by prisoners and staff on a day-to-day basis” (Jewkes, 2018: 320). We examine this question in analyzing how people imprisoned in solitary confinement and staff understand and experience the retrofitting of IMUs to include a “nature room,” and the revision of policies to prohibit punishment for self-harm and to encourage communication about unmet health needs. Through this examination, we identify specific institutional logics that thwart infrastructural reform efforts, contributing to a growing body of research examining how institutional logics within carceral settings create impediments to reforming carceral policy, practice, and beliefs (Borchert, 2016; McPherson & Sauder, 2013; Rudes et al., 2020). Specifically, we identify four institutional logics of solitary confinement – control, deprivation, mistrust, and responsibilization – and trace how these logics, combined with the “architecture of authority,” provoke the need for infrastructural reform yet simultaneously thwart the efficacy of such reforms. Together, these reform analyses offer figurative windows into understanding the limitations of infrastructural reforms within solitary confinement units.

## Methods

In this paper, we draw on (1) 183 in-depth, qualitative interviews, conducted in 2017 with both people imprisoned in and staff working in Washington state’s five all-male Intensive Management Units, and (2) systematic analysis of Washington Department of Corrections (WADOC) policy reforms, specifically pertaining to segregation practices, enacted between 2008 and 2018. The University of California, Irvine, Office of Research Institutional Review Board approved this study (HS 2016–2816), and the WADOC Research Department reviewed this approval.

### Study setting

Washington State is a mid-sized, fully-state funded prison system with the twelfth-lowest rate of incarceration in the United States (Kaeble & Cowhig, 2018). Five of Washington’s twelve prisons have an IMU building or section, which houses men in solitary confinement for an indeterminate period following one or more major rule violations. Restrictions – on commissary access, property possession, communication and visitors characterize IMU stays; people housed in these units receive approximately 10 h out-of-cell each week for yard (where the telephones are located) and showers. Stays in the IMU range from months to years (Lovell et al., 2020). Release back to the general prison population is contingent on the completion of assigned programs and avoidance of disciplinary infractions. Between 2002 and 2017, total populations in these units fluctuated from under 250 to over 600; at the time of the interviews for this study, in 2017, there were 363 people on maximum custody (the highest possible) status housed in the IMUs (Lovell et al., 2020).

The fluctuations in IMU populations at least partly reflect WADOC’s willingness to cultivate partnerships with researchers, think tanks, and advocacy organizations to develop, implement, and evaluate solitary confinement reform. These partnerships include the Mental Health Collaboration with the University of Washington (UW) in the late 1990s (Allen et al., 2001), another UW collaboration surveying the solitary confinement population in the early 2000s (Lovell, 2008; Rhodes, 2004), the collaboration with the University of [redacted] underlying the data presented here ([blinded citation)], as well as ongoing collaborations with both the Vera Institute of Justice, to reduce restrictive housing use, and Amend, to change correctional culture (Washington State Department of Corrections, 2020a; Washington State Department of Corrections, 2020b). This culture of being open to collaboration with researchers and reformers alike makes Washington an important research site for studying reforms. On the one hand, Washington arguably represents a best-case scenario of the possibilities for reform. On the other hand, reform implementation in Washington may not be generalizable to reform implementation in larger, less reform-oriented states.

### Data collection

In the summer of 2017, [redacted], the Principal Investigator, with a team of eight doctoral students, interviewed a random sample of 106 people imprisoned in solitary confinement, representing roughly one-third of the IMU population. The team identified potential interview participants from a randomly ordered list of all people housed in long-term solitary confinement at the time of interview. Researchers approached each randomly selected potential participant at their cell front to describe the study purpose and interview process. Of the 173 potential participants approached, 67 refused – a refusal rate of 39%, comparable to other studies of people imprisoned (Berzofsky & Zimmer, 2017; Calavita & Jenness, 2015; Grassian, 1983; Peterson et al., 1982).

The interviews were conducted in confidential settings within the Washington state IMUs, including visitation booths, classrooms, and offices; WADOC staff monitored these visually but not aurally monitored (ensuring participants’ privacy). Prior to the start of the interview, researchers sought informed consent, including clarifying that: participation was entirely voluntary and unaccompanied by incentives, refusing or stopping the interview would not negatively affect the participant in any way; information shared would be anonymized and protected. Participants provided oral consent to participate in the interview, and interviews were audio recorded with permission. Interviewers used a semi-structured interview instrument with 96 questions, including both yes/no and open-ended questions about conditions of daily life, perceptions about health and access to medical treatment, and experiences with programs and reforms in the IMU specifically. Interviews lasted between 45 min and 3 h, averaging just under 2 h in duration.^2^

In addition to interviews with imprisoned people, this team interviewed 77 staff members – correctional officers, supervisors, healthcare providers and social workers – working in WADOC IMUs. The research team strategically sampled staff to include staff working on each of the three daily shifts across each possible post and position (custody, non-custody medical, programming staff, and supervisors) at each IMU. Staff interviews were held in an office or other private location of the staff member’s choosing, followed a thorough informed consent process, and were audio-recorded with permission. Interviews used a semi-structured interview instrument with 87 questions, including a combination of yes/no questions and open-ended follow up questions about IMU policies, job responsibilities, personal safety, health, working relationships, and policy reforms. Interviews lasted between 30 min and 3 h, again averaging just under 2 h in duration.

All interviews completed an extensive 40-h training to learn about conditions in Washington IMUs, develop the instruments, and ensure consistency across interviewers. All interviews were recorded, transcribed, and uploaded into Atlas.ti for analysis.

### Analysis

Six team members, who had also participated in interviews, inductively analyzed the data through line-by-line open coding of a subset of interview transcripts (Charmaz, 2006; [blinded citation]). This team identified a total of 214 codes, grouped into 11 major categories (such as IMU policy, health, safety, culture). After a round of pilot coding, in which each team member completed one initial transcript coding and one re-coding, coding discrepancies were reconciled. Team members then coded within code groups of interest, such as “IMU Policy” and “IMU Conditions.” Coders met bi-weekly for 6 months to resolve discrepancies. Given this intensive, thematically-grounded process, no statistics were calculated for intercoder agreement. All quotations presented here were initially identified in the first phase of our coding process by one of the following ten (out of our initial 214) codes: nature imagery room, infractions, self-harm, suicidality, healthcare, medical care, physical health, mental illness, stigma, privacy concerns. The institutional logics that we identify in this paper emerged during our analysis of these ten codes centered on reforms and respondents’ perceptions of reform. While no respondent used the exact term of “institutional logics,” the “taken-for-granted social prescriptions” (McPherson & Sauder, 2013: 167) we identify – especially control, deprivation, mistrust, and responsibilization – surfaced repeatedly in respondents’ descriptions of the challenges or failures associated with the reforms discussed here. Where available, disconfirming evidence is provided.

In addition to identifying reforms explicitly discussed in interviews, we systematically searched the Washington Department of Corrections website for policy reforms related to solitary confinement enacted since 2008 (See Table 1 in Appendix A). The three reforms we discuss here are among dozens identified in this process.

### Study sample

Our random sample of 106 participants imprisoned in the IMUs had a mean age of 35; an average IMU stay of 14.5 months; and a mean of 5 prior convictions resulting in prison sentences. Of our incarcerated participants, 42% identified as white, 12% as African American, 23% as Latino, and 23% as another race or ethnicity. There were no significant differences between our random sample and the total IMU population at the time of interviews. However, our participants were younger, serving longer sentences, more likely to be gang-affiliated, more likely to be Latino, and had more violent criminal histories than the general prison population ([redacted citation]). Among the 77 staff that participated in interviews: 74% were male, 84% were white, 57% were married, and the average age was 42. Overall demographic data for WADOC staff or those stationed in the five IMUs were not available, so we are unable to compare the staff participant demographics with overall demographics of IMU or WADOC staff.

## Results

In the following sub-sections, we discuss three types of infrastructural reforms that the Washington DOC has implemented to address, and ideally reduce, the mental and physical harms of solitary confinement: nature rooms, routine suicide watch, and wellness checks. After describing when and how each reform was implemented, we discuss how both people imprisoned in IMUs and staff working in these units interpreted and experienced such reforms, and particularly how the built environment interacts with the institutional logics of control, deprivation, mistrust, and responsibilization, ultimately limiting both the uptake and the potential benefits of such reforms.

### Screening windows

Nalini Nadkarni, a forest ecologist, first proposed the idea of a nature imagery room, or a “blue room,” in a TED Talk in 2010. Nadkarini (2010) argued that exposing people imprisoned in solitary confinement – who spend months, and sometimes years, moving only between the barren concrete of small cells and caged exercise yards – to images of nature could have a calming, restorative effect. She first tried to implement the idea of a blue room in Washington state IMUs, but faced resistance from staff on the designated trial unit, and dropped the idea. Oregon prison officials approached her in 2013 and implemented the idea in an IMU at the Snake River Correctional facility: during the first year of the blue room’s operation, the number of disciplinary referrals fell significantly in the unit where people had access to the blue room (McCoy, 2015). In 2015, just 2 years before data collection for this project began, Washington implemented its first blue room in an IMU at Washington Corrections Center (Correctional News, 2015). As a result, we directly asked all IMU staff and probed imprisoned interviewees to describe what they knew about the blue room and what they thought about it.

Though the exact set-up of these rooms varies by facility, their basic features include a chair, a flat screen TV, and a nature video playing on that TV. By offering people imprisoned in solitary confinement a “screening window” into nature, blue rooms represent an explicit effort to change how the IMU’s built environment is experienced by imprisoned people and staff alike. Although, at the time of our interviews, three prisons had implemented blue rooms, very few respondents were familiar with them. This limited awareness of the existence of the blue rooms provides an initial indication of their limited effectiveness, either as a helpful resource for individuals imprisoned in solitary confinement, or as a transformative intervention re-structuring the institutional culture of solitary confinement. Indeed, respondents who were aware of the blue rooms were overwhelmingly negative in their evaluations of the intervention. Austin,^3^ a sergeant on an IMU, succinctly described the blue room as a “failed policy.” But why? Those respondents familiar with the blue room identified two infrastructural obstacles to successful implementation of the reform: the physical constraints of architecture and the cultural constraints of institutional logics of control, mistrust, deprivation, and responsibilization.

First, finding a physical space within the IMU that could be “normalized” (Crewe, 2020) via the introduction of nature (albeit through a screen) proved an impossible challenge for some facilities. For example, Patrick, a correctional unit supervisor (CUS) at one facility, explained that the unit had not implemented a blue room due to space limitations: “We would have to designate a cell or something for that, and we haven’t done that yet. And I don’t think they would ever sacrifice. .. a cell for it because they’re needed.” On the surface, the primary impediment to creating a blue room was described as infrastructural: carving out even a virtual window into nature from the concrete blocks of the IMU was out of the question. In this way, the built environment thwarts the potential of normalizing reforms (Crewe, 2020; Reiter et al., 2018).

Second, the institutional logics of control dominating the IMU further undercut efforts at normalizing the built environment of the IMU. Joaquin, the one person among the 106 imprisoned interview subjects who had ever used a blue room, explained how the experience of being controlled eclipsed any experience of “nature” in the room:

[T] hey take about 3 to 5 min just to like fully strap you to the thing. And I even told the C.O., man, I feel like I’m going to get the lethal injection or something because you’re getting like – like your feet are tied up, and your hands are tied up, and you’re tied up to the chair. It’s like really uncomfortable.”

Once strapped in, Joaquin watched a nature video that the custody officer (C.O.) had selected for him from a short list: *Moose in the Lake*, featuring a five-minute loop where a moose, standing in a lake, shakes his ears and tail, birds chirping in the background. His overall assessment of the experience: “horrible”. For Joaquin and for staff, the governing institutional logic of the IMU as a space to control people, not as a space to screen nature videos, undermined attempts to remediate the barrenness of the environment.

For custody staff, too, the ordeal associated with transporting people imprisoned in solitary confinement to and from the blue room ranked as their top complaint about the policy. In fact, Kevin, a member of the custody staff, described imprisoned people’s requests to go to the blue room as not only disruptive to the daily routine of the IMU, but as calculated to “stop our deal”: “If they want to piss us off – not me specifically, but us as a group – they’ll say, ‘I want to go to the nature imagery room. I need to do this. I’m feeling suicidal.’ And then they’ll go to the blue room, and they watch trees and stuff.” Kevin acknowledged that the blue room might “actually calm” people with “legit, honest mental health issues,” but for “90% of the guys in segregation,” requesting time in the blue room was new a weapon to wield against staff, disrupting their routines, and challenging (but not changing or softening) the institutional logic of control governing the IMU. Kevin’s perspective also reveals how the institutional logic of mistrust – interpreting any imprisoned person’s request, even one to exercise access to a new resource, as manipulative – thwarted access to the blue room. Indeed, in order to minimize this disruption to IMU routines, custody staff usually required people to choose between going to the indoor blue room to “see” nature or going outside to a concrete exercise yard, stripped of nature. “Choosing” the blue room, then, reinforced both the institutional logics of responsbilization (forcing the imprisoned person to choose, albeit among limited options, to take care of themselves) and deprivation (giving up limited time outside).

Steve, a hearings officer, was the only staff participant to express any enthusiasm for the blue room. He stated, “I really – I like the concept, and I’m – I’m on board with it.” However, while he supported the theoretical idea, Steve’s thoughts on the reality of the blue room echo Austin’s description of it as a “failed policy.” Steve explains, “I think that we jumped in and created the policy for it. We kind of rushed it a little bit I think; unfortunately, there wasn’t enough input from the boots on the ground.”

Analyzing the attempted implementation of blue rooms in the Washington IMUs offers a window into how the constant of control – over both movement and choice – pervades the IMU and thwarts reform efforts. The largely negative descriptions of the blue room as a failed reform reflect both structural impediments and the institutional logics of control and deprivation that are inherent to the IMU. In the end, attempts to integrate the softness of nature into the hardness of the concrete IMU reaffirmed the institutional imperative to manage and be managed.

### Watch windows

In another effort to respond to critiques of solitary confinement as harmful, Washington DOC implemented a series of policies in 2014 to limit and more humanely respond to self-harm in solitary confinement: eliminating disciplinary infractions for people who attempt to harm themselves while in solitary confinement and reacting, instead, by moving them into suicide watch cells (Jenkins, 2014; Washington Department of Corrections, 2020d; Washington Department of Corrections, 2020e). These reforms responded to a growing body of research documenting the association between solitary confinement and high rates of deliberate self-harm and suicidality on the one hand (Kaba et al., 2014; Reiter et al., 2020), and a persistent correctional mindset that people imprisoned in solitary confinement who engage in self-harm do so merely to obtain a desired response, or to exercise control over an oppressive environment (often called simply “manipulation”) (Dear et al., 2000; Groves, 2004; Kenning et al., 2010). This correctional mindset – another example of the cultural logic of mistrust coexisting with the cultural logics of control, deprivation, and responsibilization highlighted above – shapes staff treatment of and responses to self-harm. Staff attempt to avoid rewarding “manipulators” while providing treatment to those in “genuine” need. By eliminating punishments and establishing clear policies for responding to self-harming behavior, Washington officials sought to avoid this process of sorting people imprisoned in solitary confinement into trustworthy and untrustworthy mental need categories. To evaluate how these policies were being interpreted and implemented, we asked all interviewees imprisoned in the IMU whether they had been punished or thought they would be punished for engaging in self-harm, and we asked staff general questions about IMU reforms and their experiences using restraints on people imprisoned in the IMU.

Some participants said they would not be punished for engaging in self-harm, as Caleb explained: “They used to punish us, but they stopped – they stopped infracting us for self-harm now.” However, the overwhelming majority of participants disagreed, believing instead that self-harm would still be met with some form of punishment. Bleakly, another participant named Luis stated: “If you try to harm yourself then you better do it right because otherwise, they’re going to punish you for it.” One such response to self-harm and suicidality that was still perceived and experienced as punishment by our respondents was “suicide watch,” in part because the experience of being “on watch” reinforced institutional logics of deprivation.

Under Washington’s suicide-response reforms, incarcerated people who report feeling suicidal, engage in “genuine” self-harm, or are deemed a suicide risk by clinical or security staff are placed on suicide watch. While on watch, the person is removed from their cell and placed in an observation room with no property and minimal (if any) clothing. If they are not entirely naked, people on suicide watch are given a thick “suicide smock,” also referred to as a “turtle suit,” “green suit,” or as one respondent described it, a “human-sized potholder.” Unlike typical IMU cells, suicide watch cells have a large window and/or a camera that allow staff to monitor its occupant at all times. One participant described the process of suicide watch:

They take everything that you have in your cell, and I think the first 24 h they put you in a holding cell. It’s pretty much as big as this [visitor booth] but without the desk and a stool. And you’re in there just butt naked and that’s the punishment. Then they take you back to your old cell. You don’t have anything. You don’t have no mattress, no linen, anything that you might be able to hang yourself with or anything sharp or whatever. Just recently somebody was acting out, and they put him out their cell naked. I mean, that’s pretty humiliating.

The humiliation of being transported to and from the observation cell contributed to the perception that suicide watch was a punitive, not a therapeutic, response to severe mental distress. During this “walk of shame,” people on suicide watch are not only on display in front of staff from the observation cell, they also become a spectacle for all residents of the housing unit to see. Aside from functioning to incapacitate, extracting a person from their “house” and putting them on the display of suicide watch seems unlikely to resolve suicidal ideation, especially if there is no improvement in the material or social conditions of deprivation that may have induced self-harming behaviors in the first place. Rather than stop the potential for self-harm, suicide watch may actually compound the harms of solitary confinement by introducing yet another form of dehumanization in the service of “care.”

Although several imprisoned participants reported prior near-lethal suicide attempts, which landed them in “outside” hospitals, where they received adequate care, most imprisoned participants who described experiencing suicidal ideation in the IMU landed on suicide watch, behind the “watch window.” Participants’ experiences of suicide watch call into question the intended purposes of moving people imprisoned in the IMU from one extreme form of isolation to another (even more extreme) form of deprivation. Removing any possessions, while eliminating the potential for certain forms of self-inflicted harm, also removes small comforts like books, radios, and writing materials that might otherwise be an essential aid in coping. Placement on suicide watch also moves the person away from their neighbors, who often serve as one of the few social supports in near total isolation. Instead, the person on suicide watch is left with nothing but more time and silence in which to ruminate, which poses a risk of exacerbating, rather than relieving, their mental health crisis. And yet, within the context of suicide watch, total isolation and material deprivation are institutionally framed as forms of care, rather than harm. Put differently, despite the department’s intentions to respond to self-harm and suicidal ideation in a less punitive fashion, institutional imperatives around risk management and institutional logics of deprivation, as well as mistrust, appeared to undermine the legitimacy of the reform as an actual form of care.

### Drive thru windows

In a third effort to respond to critiques of solitary confinement as harmful, Washington DOC implemented a cluster of policies to increase imprisoned people’s access to medical and mental health staff and care in restrictive housing units: increasing the number of mental health staff, shortening response times for “call out requests” to be seen by a healthcare provider, and requiring immediate responses to mental health emergencies. The most consistent way Washington DOC sought to ensure access to healthcare in the IMU: requiring a non-custody staff member (with some healthcare training) to conduct daily “wellness” checks for each person imprisoned in the IMU (Washington State Department of Corrections, 2020c). During these wellness checks, staff look into each person’s cell for signs of “ADLs,” or Activities of Daily Living. The interactions are usually brief, at the cell front, prioritizing efficiency – much like a fast-food drive thru, versus a dine-in restaurant experience. There is very little time spent at each cell, and a quick verbal exchange satisfies the requirement of observing ADLs. Solitary confinement expert Craig Haney has criticized these cell front checks, both because the policy presumes that “whether or not someone is suffering from mental illness [can be established] merely by looking at them” and because the checks substitute “perfunctorily ask [ing] a superficial question or two” for “meaningful mental health observation, assessment, or contact” (Haney, 2017, 317). In order to better understand exactly how people imprisoned in solitary confinement and staff experienced these daily cell front checks, we asked respondents specifically whether or not they had ever attempted to access medical or mental health care, and why or why not.

In this context, our interviewees described their interactions with mental and physical health care staff via the cell front window. As respondent Eli explained: “Oh, they do wellness checks every day. See if you’re alive and all right … Come by their house. If you’re not moving, they’ll – “Hey, we need to see you moving.” You’ll move. They’ll be like, “Okay, thanks. ‘Bye!” Other respondents characterized these “cruise by”^4^ wellness checks as superficial and not worthwhile, with little to no opportunity for meaningful interaction. Some people imprisoned in solitary confinement appreciated this brevity, however; they wanted no association with medical or mental health staff, who were synonymous with mental illness, and weakness. Rob described this when discussing how often he saw a mental health counselor: “Most of us don’t like to see them … because it’s all related to the medication and the … Dings. You call it. It might be a bad word.” In other words, an institutional logic that mental health care is stigmatizing created a barrier to accessing care, especially at cell front. This echoes the intensity of the shame respondents described experiencing on display on the way to suicide watch.

A second institutional logic endemic in prisons, and especially IMUs – responsibilization (Crewe, 2007; Garland, 2001; Sexton, 2015) – created another barrier to accessing care. Specifically, staff expected people imprisoned in the IMU to initiate any engagement with mental health staff, beyond the minimum, daily signs of life check. As staff member Austin explained, in describing the purpose of the daily wellness checks: “Primarily, again, I’m just making sure that they’re all alive. And if they have any questions, then they can ask me when I’m doing my check, and stuff like that. So, I’ll stop and talk to them then.” Ultimately, this kind of drive-thru engagement puts the responsibility of care on people imprisoned in solitary confinement – they must interrupt the staff members’ efficiency-focused rounds, ask for care above the minimum wellness check for signs of life, and also potentially put themselves at risk of social stigma merely by associating with a mental health care provider.

Indeed, cell front healthcare lacks confidentiality, creating privacy and safety concerns among people imprisoned in solitary confinement. Conversations at these cell front windows are visible to all and can be overheard by people in neighboring cells. Cell doors are made of thick steel and people imprisoned within them must yell loudly in order to be heard by staff, compromising their own privacy. As respondent Tyler described:

They’re asking people on the tier, “What’s wrong with you?” And people are talking to them. So, guess what, I just know everything about this guy, what meds he’s on, what’s he going to do, how psycho he is. […] Someone needs to tell them, hey, you can’t just go to the door and say, you know, let’s have full conversations through a freaking door when the whole tier is listening. I don’t want to talk about my problems if they’re all going, “Oh, Tyler’s a nut case.” They’re going to say that, and they’re going to start a cell war, or start cell banging.

Respondents described how this discomfort with having their cell-front conversations broadcast extended to receiving any form of care, even prescribed medications. As in lower custody units, medical staff dispense medication directly to people imprisoned in segregation. Unlike other units, however, people imprisoned in the IMU cannot queue up in pill line, so pill line happens on the tier and at each cell front. Staff, too, noticed the discomfort people imprisoned in IMUs experienced from the lack of medical privacy; as Kelly explains:

I don’t know what the guys on the tiers actually talk to each other about. But I do know that some of the guys that have refused in the past, it is basically because somebody near them has said, “Oh, you take ding biscuits, oh.” […] And I have had numerous guys that are just like, we get them on a medication because of their behaviors, and they’ll be on it for a little bit, and then they decide, “Uh-uh, no way, mm-mm.” And then you kind of look at who’s near them, and it’s kind of like, “Okay, so, yeah, I think you’re being talked out of this.”

Cell-front care compromises the little privacy that people imprisoned in the IMU have, leaving them vulnerable, as other imprisoned people or staff may use their sensitive information against them. Particularly in the IMU, people with mental illness are stigmatized and relentlessly harassed (e.g., called “dings”). The ding characterization can follow people imprisoned in solitary confinement back into lower custody units upon release from segregation.

In addition to privacy concerns over mental health care, the broad visibility and lack of privacy associated with cell-front health care also affects whether or not a person imprisoned in solitary confinement seeks treatment for sensitive health concerns. Respondents reported being embarrassed by cell-front interactions, where they were expected to communicate their physical, mental, and emotional symptoms publicly to staff at their cell front. For example, Luke expressed:

You’re pretty much being asked in front of everybody on the tier … I’m depressed … and I’m a wreck right now, and I’m not doing good. Instead of saying that, you’re like, no, I’m fine. Because you don’t want other people on the tier.. .. knowing your business and what’s going on with you. It’d be embarrassing. You don’t want to be made fun of and peer pressure.

These visits function to put the person on display; a captive audience watches during the physical or mental health consultation with a provider. With only a few minutes to communicate their needs, this interaction can be pressured and stressful, especially when that provider is a woman. For example, Andrew worried that others on the unit would think he was “trying to flirt” because he was trying to explain his healthcare needs while also being “a gentleman.” These interactions are highly scrutinized by both staff and other people imprisoned in the IMU, a short cell-front interaction can be interpreted not only as a veiled attempt at flirtation, as Andrew worried, but as manipulation. Further, these interactions can also be viewed as violations of social code among other people imprisoned in solitary confinement. Isaac, for example, said that he declined offered mental health services because of the “politics” that dictate “you can’t talk to them.”

Against these pressures, our respondents also described how cell-front embarrassment deterred them from seeking out care by, for example, sending a medical kite requesting a visit. Here, Marco explained:

And if they do come and see you, what they do is this embarrassment treatment. .. they’ll come over to your door and you explain everything that’s going on in front of your door. And they say it really loud, so everybody can hear. So the nurse will come over to your door, ‘Hey, he’s got a giant hemorrhoid with this-and-this.’ They’ll say it on the tier; let everybody know that you’re having problems with that. And then everybody knows your whole medical situation. And so if you’re having diarrhea, […] herpes – they’re putting it all on the tier!

Some interviewees attributed this practice to a lack of awareness among medical and mental health staff, while other participants, like Marco, perceived it as an intentional strategy to deter people imprisoned in the IMU from requesting health care – deterrence via embarrassment.

In this way, institutional logics of mistrust (between imprisoned people, as well as between imprisoned people and staff), coupled with limitations of the physical environment of the prison (an absence of private spaces), undermined efforts to improve healthcare. As people imprisoned in these units are confined to their cells and unable to move about the unit without a two-man escort, each imprisoned person’s 12 × 8-in. cell front window serves as their portal to receive and ask for patient care. Again, the reality of the restrictive structural environment of segregation units and the institutional logics that pervade them undermine the efficacy and reach of efforts to improve the prison environment.

## Discussion

By drawing upon the direct experiences of people imprisoned in solitary confinement and staff working in these units, our findings demonstrate how both institutional logics and physical infrastructure thwart the efficacy of reforming solitary confinement. Space constraints in IMUs interact with institutional logics of control, deprivation, and responsibilization and ultimately impede the most modest attempts at transforming the inherently harsh practice of solitary confinement. A fourth institutional logic – pervasive mistrust among prisoners and between prisoners and staff – created additional barriers to accessing the resources reforms attempted to provide (e.g., mental health care).

Our analysis of attempts to implement screening, watch, and drive thru window reforms reveals how institutional logics are actually reinforced under incrementalist reforms. In fact, the reforms we analyze, like those Rudes et al. analyzed in their study of PREA implementation (Rudes et al., 2020), created new forms of tension between people imprisoned in solitary confinement and correctional staff. Staff perceived requests to use the nature imagery room as threats to unit routines, and suicide watch and cell-front checks introduced new costs for imprisoned people seeking mental and physical health services.

Experiences of IMU policy reforms also take place within a particularly harsh and inflexible physical environment – cells with few actual windows and thick steel doors, where any sound reverberates, dissolving any hope of privacy. The physical design of solitary confinement impedes implementation of reforms to: secure physical space for a nature imagery room, provide humane surveillance for those engaging in self-harm, and encourage discussion of health concerns (without any privacy). Reforms meant to promote therapeutic comfort, care, and wellness, flounder within a physical environment designed to control human bodies and deny meaningful social contact. Here, our findings help to shed light on the critical role of environmental capacity in reducing the harm not just of incarceration generally (Jewkes, 2018), but of solitary confinement specifically.

Taken together, our findings suggest that, despite intentions to improve conditions of confinement, the aforementioned reforms operate and are perceived as window dressing. Extreme isolation is made possible through repressive spaces that are both physically and culturally (through entrenched institutional logics) resistant to change. Virtual simulations of the natural world and reactive and short-lived social encounters through suicide watch windows and cell front checks ultimately fail in practice because they do not seek to transform the institutional logics (or legal templates) that define the practice of solitary confinement, including the fundamental deprivation of liberty in response to perceived risk. Regardless of growing recognition that people imprisoned in solitary confinement require adequate care to prevent mental and physical deterioration, staff perceive people imprisoned in the IMU as persistent threats and purposeful manipulators. This logic of mistrust inherently conflicts with incrementalist reforms that hinge upon a logic of responsibilization, requiring people imprisoned in solitary confinement to advocate for and assert themselves – whether that’s *choosing* to go to the blue room instead of yard, *choosing* to speak to a mental health staff at your cell front, or even *choosing* to self-harm as a means to “feel” something beyond one’s own isolation. Staff agency – including to believe or not believe a person imprisoned in solitary confinement, or to see a person imprisoned in IMU as worthy of care as opposed to deserving only of punishment – is, thus, pitted against imprisoned people’s agency and their decisions to take “advantage” of reform efforts or not.

Just as Rudes et al., (2020) argue that successful PREA implementation requires that staff ascribe to specific values regarding gender, sexuality, consent and assault, so too does successful implementation and expansion of solitary confinement reforms require a shift in values. Namely, this shift represents a humanizing of prisoners – even those deemed to be the “worst of the worst” – as worthy of dignity, social contact, due process, and more. And while scholars have suggested that the contradiction between custody and care can precipitate new institutional logics capable of resolving organizational misalignment within prison (Marti et al., 2017; Thorton et al., 2012), our study illustrates the inertia of deprivation imperatives within solitary confinement. This suggests that some legal templates of punishment, such as isolation, might be particularly resistant and maladaptive to reformist efforts.

## Conclusions

By examining how incrementalist reforms are mobilized in a structure of extreme confinement like the IMU, and how they are experienced by both people imprisoned in solitary confinement and staff working in these units, we show how the transformative potential of these reforms is eclipsed. As our participants have described, the blue room, suicide watch, and cell-front checks generate new conflicts between people imprisoned in solitary confinement and correctional staff, which only add to the strain and drain of solitary confinement. A room designed for relaxation feels like an execution, expressing suicidal ideation results in shame and indignity, and interventions meant to make healthcare more accessible inadvertently encourage its avoidance.

Questions of futility and ineffectiveness aside, what we have framed as *windows* of reform demonstrate the significance of penal aesthetics and geography in thinking about contemporary punishment and the movement to either reform or abolish solitary confinement. Not only do incrementalist reforms reflect a particular re-imagining of solitary confinement – that it can become a caring and therapeutic space – but, through their implementation, they seemingly attempt to dull the severity of isolation. Accordingly, within an incrementalist perspective, a softened prison aesthetic is at least preferable to a hardened one. What if, however, environmental softening of the prison only produces different forms of harm, as opposed to actually reducing pain and suffering?

As Crewe offers in his re-theorization of the pains of imprisonment, power in prison is experienced “as both firm and soft, oppressive yet also light. It does not so much weigh down on prisoners and suppress them as wrap them up, smother them and incite them to conduct themselves in particular ways” (Crewe, 2011, pg. 522). Indeed, our windows of reform put on display this interaction between environment and personal conduct. People imprisoned in solitary confinement are given the *option* to decide on what nature images to watch, to disclose if they are considering self-harm, or to interact with a health professional within earshot of others. Providing these options may momentarily disrupt the monotony of solitary or intervene upon an immediate mental health crisis, yet these options ultimately require imprisoned people to conduct themselves as manageable and docile individuals in the face of institutional logics framing them as threatening and manipulative. All the while, people imprisoned in solitary confinement are still spending nearly 22 h in their cell, alone, where physical and mental harms can continue to compound. As such, infrastructural and incrementalist reforms like those discussed in this paper may, instead, reinforce the practice of solitary by seemingly softening its edges, thereby making it all the more difficult to fundamentally change, let alone dismantle (Pifer, 2016; Reiter, 2012).

Without deeper consideration of and engagement with the institutional logics that govern solitary confinement, these efforts to reform or “soften” solitary confinement become superficial “window dressing.” The (intentional or unintentional) superficiality of solitary confinement reforms raises a larger question: whether attempts to reform solitary confinement – as opposed to do away with the practice entirely – within the context of mass incarceration reform can be anything other than superficial. The very emergence of the modern-day prison is itself a product of penal reform, where incrementalist reforms shifted the shape of the prison, the fundamental nature and mechanics remained unchanged. Perhaps, given this especially complicated nature of prison reform generally, and solitary confinement specifically, any attempt to reform solitary confinement necessarily can only be window dressing.

Despite the rather bleak characterization of reform efforts described by staff and people imprisoned in solitary confinement, this is not to say that efforts aimed at minimizing the harms of solitary confinement are for naught. The only way to learn whether a reform has succeeded or failed is to implement it – and then allow for analysis. This speaks to whether prison administrations are reform-oriented or reform-adverse, which is a crucial distinction in understanding the successes of and lesson learned from incrementalist reforms. Even the most well-intentioned reforms, like those attempted by the Washington DOC, should be scrutinized in order to determine if they are producing the desired outcomes or instead, reproducing a different, but nonetheless damaging set of harms to people imprisoned in solitary confinement. Further, even well-intentioned reforms are often stymied by the underlying institutional logics and norms of restrictive housing spaces, and efforts to directly address culture may serve to create positive change in ways that reforms focused on policy or built environment alone cannot. One of Washington state’s most recent reform efforts confronts this issue of culture, through a partnership with Amend, an organization that uses a public health approach to change correctional culture (Amend, 2020). Perhaps this effort will aid in dismantling reform-resistant cultural norms and institutional logics within solitary confinement spaces, ultimately making meaningful infrastructural and policy change possible. Still, in order to understand the possibilities and limits of reform, further research is needed to examine how everyday actors within prison settings either reconcile or challenge demands for reform with the institutional logics that animate carceral spaces.

## Data Availability

Data cannot be shared publicly due to highly sensitive and ethical considerations. Data pertains to prisoners’ experiences and other identifying information. None of the data was approved to be shared outside of the research team. The authors of this paper are happy to provide supplemental material containing additional quotations if deemed necessary by Health & Justice.

## Appendix

**Table 1:** Type of WADOC Restrictive Housing Reform

Conditions of Confinement	Behavior Modification	Mental Health	Preventative	Organizational Restructuring
Congregate Programming	Cognitive Behavioral Therapy (in-cell)	Elimination of self-harm infractions	Alternative sanctions	Creation of a Mission Housing Administrator
Level System	Individual Behavior Management Program (IBMP)	Disruptive Hygiene protocol	Alternative Specialized Housing Units (TRU, WRU)	Mission-Based Housing Units & Teams
Elective programming (GED, Redemption, Book Club)	Chemical dependency class	Increased access to counselors, MH staff		Facility Risk Management Teams
Nature Immersion (Blue) Room	Transition/Step-down Unit			Indeterminate sentencing

## Footnotes

1 The field of public health has moved toward the use of people-first language to preserve the humanity and dignity of all populations. In this piece, we have chosen to use variations of “people imprisoned in solitary confinement” (rather than alternatives such as “people who are incarcerated”) as a way to acknowledge the involuntary nature of their confinement and to prevent sanitizing the harms experienced during prolonged incarceration and segregation.

2 One-year follow-up interviews were conducted with all participants who were still incarcerated in the summer of 2018 (*n* = 80), but this paper relies only on data from the 2017 interviews, as those interview instruments more specifically addressed the relevant reforms.

3 Pseudonyms have been assigned to all interview participants to ensure anonymity.

4 Quotation from respondent Tyler.
