# Correction to: Window dressing: possibilities and limitations of incremental changes in solitary confinement

**DOI:** 10.1186/s40352-021-00152-8

**Published:** 2021-09-14

**Authors:** Dallas Augustine, Melissa Barragan, Kelsie Chesnut, Natalie A. Pifer, Keramet Reiter, Justin D. Strong

**Affiliations:** 1 Department of Medicine, University of California, San Francisco, USA; 2 Department of Sociology, California State Polytechnic University at Pomona, Pomona, USA; 3 Vera Institute of Justice, Brooklyn, USA; 4 Department of Criminology and Criminal Justice, University of Rhode Island, Kingston, USA; 5 Department of Criminology, Law & Society, University of California, Irvine, USA

**Correction to: Health Justice 9, 21 (2021)**

**https://doi.org/10.1186/s40352-021-00145-7**

Following the publication of the original article [1] the authors noticed that the published version of the manuscript has redacted and blinded citations.

The original article [1] has been updated.
